# Zr Incorporation Into Lithium Nickel Oxides: Bulk or Surface Substitution?

**DOI:** 10.1002/advs.78453

**Published:** 2026-11-04

**Authors:** Lars Winkler, Geon‐Tae Park, Liang‐Yin Kuo, Martin Ihrig, Konstantin Köster, Johan E. ten Elshof, Mark Huijben, Myoung‐Chan Kim, Min‐Gyu Seo, Yang‐Kook Sun, Payam Kaghazchi

**Affiliations:** ^1^ Institute of Energy Materials and Devices (IMD) – IMD‐2: Materials Synthesis and Processing Forschungszentrum Jülich GmbH Jülich Germany; ^2^ MESA+ Institute for Nanotechnology University of Twente Enschede The Netherlands; ^3^ Department of Energy Engineering Hanyang University Seoul South Korea; ^4^ Chair For Theoretical Physics VII and Bavarian Center For Battery Technologies University of Bayreuth Bayreuth Germany; ^5^ Department of Chemical Engineering National Taiwan University of Science and Technology Taipei City Taiwan

**Keywords:** Ab Initio Calculations, Cathode Materials, Cation Ordering, Doping, Li‐ion Batteries

## Abstract

High‐capacity lithium nickel oxides (LNO) are promising cathode active materials for next‐generation batteries. However, their practical application is limited by poor cycling stability. Doping the LNO cathode with high‐valence elements is an effective strategy to enhance structural stability. In this study, we investigate the mechanism of Zr^4+^ incorporation into LNO using experimental and theoretical techniques. Our findings reveal that under oxygen‐rich conditions, Zr‐doped LNO can be realized as a single phase, but most Zr cations localize at the surface of primary particles. In contrast, under oxygen‐deficient or non‐equilibrium conditions a secondary layered Li_1‐x_Ni_1+x_O_2_ phase, characterized by Ni migration into the Li layers (cation ordering), is stabilized coexisting with Zr‐doped LNO. This promotes elongation of primary particles at the aggregation surface. The Zr enrichment at the microparticle surface, Zr‐induced cation‐ordered superstructure, and the formation of elongated grains enhance the chemical stability as well as the structural integrity at atomistic‐ and micro‐scale of the LNO cathode, improving its cycling performance. However, only 1–3% Zr is necessary to maintain the initial capacity and enhance cyclability through surface coating and low bulk doping with Zr. With increasing Zr concentration, an electronically insulating impurity (Li_2_ZrO_3_) forms, leading to a decrease in the initial capacity.

## Graphical abstract

Mechanism of Zr incorporation into lithium nickel oxide (LNO) cathodes is studied from atomistic to macroscale. Zr doping enhances cyclability of Li‐ion batteries with LNO via bulk, interface, and grain boundary optimization leading to structural integrity at atomistic‐ and micro‐scale of secondary particle aggregates. However, excess Zr doping leads to insulating impurity phases limiting initial capacity.

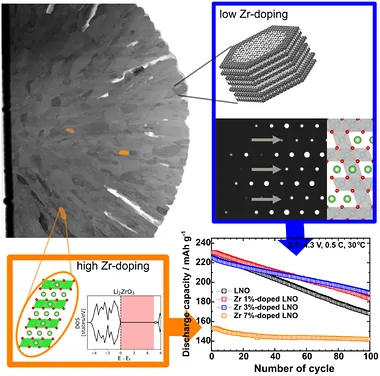

## 1 Introduction

High‐capacity lithium‐based nickel oxides (LNO) are potentially very attractive cathode active materials for the next‐generation electric vehicles [1]. They can theoretically provide gravitational and volumetric capacity up to 275 mAh/g and 1300 mAh/cm^3^, respectively, with an operating voltage of around 4.2 V vs Li/Li^+^ [1, 2, 3, 4]. Moreover, they show an acceptable low‐temperature performance [5]. However, Ni‐rich layered cathode materials, such as LNO suffer from cycling instability, which can be due to microcracking of its particles, electrolyte decomposition on its surface and formation of thick insulator cathode electrolyte interphase (CEI) layers as well as Li/Ni interchange and oxygen loss [3, 6, 7, 8, 9, 10]. For example, Yoon et al. showed that LiNiO_2_ exhibited stable cycling performance at 4.1 V, maintaining 95% of its initial capacity (164 mAh/g) after 100 cycles, while operation at higher voltages of 4.2 and 4.3 V led to accelerated capacity degradation, with only 81% of 191 mAh/g and 75% of 232 mAh/g achieved, respectively [3].

Beside the tendency of lithium nickel oxide to form Li_1‐_ *_x_* Ni_1+_ *_x_* O_2_, with Ni on the Li sites rather than LiNiO_2_, it is well known that during cycling the structure experiences phase transitions and lattice distortions that promotes migration of Ni ions from the transition metal (TM) sites toward the Li sites, even for stoichiometric compositions of LNO [1, 2, 11, 12]. In the same time some Li ions occupy vacant Ni positions at TM sites. This behavior, which is known as Li/Ni interchange (or cation mixing), can hinder Li transport and/or decrease initial capacity [13, 14, 15]. In special cases the cations (Ni) occupy the Li sites in an ordered manner, known as cation ordering, which can stabilize the cathode crystal structure and improve the capacity retention [16, 17]. If Ni vacancies form during synthesis or due to charging/discharging, not only the valency of other Ni‐ions have to change for charge compensation, it can activate the oxidation of oxygen anions from O^2−^ to O^2n−^ and the associated oxygen release, which can lead to chemical and mechanical instabilities [9, 15].

Elemental doping and/or surface coating are commonly‐used strategies to prevent the aforementioned problems in Li*_x_* Ni*_y_* O*_z_* and other Ni‐rich cathodes [18, 19, 20, 21]. Dopants such as Mg^2+^ (0.72 Å) and Al^3+^ (0.54 Å) have been found to reduce Li/Ni mixing of LMO_2_ (M = Ni, Co & Mn and/or Al) by either changing the oxidation states of Ni from 2+ to 4+ and 3+ or, in case of Al^3+^, by strongly bonding with oxygen to suppress oxygen release and stabilize the lattice structure, reducing Ni‐migration into Li sites [22, 23]. Moreover, Mg^2+^ can occupy Li sites and thus block vacant Li sites for Ni^2+^ [23]. It has been found that doping of a Ni‐rich NCA cathode with B^3+^ promotes the formation of a Li_3_BO_3_ surface coating layer on its (003) surfaces, which strongly alters the microstructure of the material and reduces mechanical stress and microcracking [24]. The Li_3_BO_3_ layer can also alleviate electrolyte decomposition on the cathode surface.

Another type of promising dopant are high‐valent transition metal cations, such as Zr^4+^ (0.72 Å) which have a similar ionic radius as Mg^2+^. Choi et al. found Zr‐doping of NCM622 suppresses the Li/Ni mixing facilitating Li^+^ diffusion [25]. They also found that Zr^4+^ dopants alleviate microcracks along grain boundaries for Li[Ni_0.6_Co_0.2_Mn_0.2_]_TM_O_2_ (NCM622) which increases the cycling stability. However, Han et al. found an increased Ni^2+^ concentration within Zr^4+^‐substituted Li[Ni_0.8_Co_0.1_Mn_0.1_]_TM_O_2_ (NCM811) which ultimately would foster the Li/Ni mixing [26]. Gao et al. analyzed impurities and found a formation of Li_2_ZrO_3_ coating layer on 1% Zr‐doped NCM811 [27]. Incorporation of Zr into Ni‐rich layered oxide cathode materials can, therefore, lead to bulk Zr doping and/or surface coating with zirconate depending on precursor concentrations and experimental conditions (temperature, partial pressure of surrounding or applying gases). Generally, experimental studies find that applying Zr doping and/or Li_2_ZrO_3_ coating improve the electrochemical performance, in particular cyclability, of Ni‐based layered oxide cathodes [25, 26, 27, 28].

In this study, we characterize the mechanism of Zr incorporation into LiNiO_2_ and its influence on the performance of cathode by combining various experimental and theoretical techniques. Systematic experiments with varying Zr contents were conducted validating theoretical predictions on the doping mechanism. Despite the relatively high Zr concentrations studied in this work, we use the term “doping” because it is widely adopted in the battery materials field.

## 2 Results and Discussion

To prepare Zr‐doped LNO, we employed the dry doping method, where the Zr source was introduced during the calcination process. Specifically, Zr was added as ZrO_2_ by mixing it with the Ni(OH)_2_ precursor and LiOH prior to high‐temperature calcination, ultimately yielding Zr dry‐doped LNO. Wet doping is generally more effective for incorporating Zr into the crystal structure of LNO, as Zr is readily integrated during the growth of Ni(OH)_2_ precursor particles. In comparison, in the dry doping method, Zr is introduced externally to the Ni(OH)_2_ precursors, resulting in its preferential localization near the surface of the cathode grains. These distinct doping methods lead to differences in both the physicochemical properties and electrochemical performances of the cathode materials. Based on the electrochemical performance comparison (Figure A1 in SI), the Zr dry‐doped cathode exhibited higher capacity compared to the Zr wet‐doped cathode. Although not all Zr concentrations were experimentally compared using both synthesis methods, the observed trend suggests that the inhomogeneous distribution of Zr (i.e. segregation of Zr to interfaces and grain boundaries, which will be discussed later) and the resulting evolution of distinct physicochemical characteristics contribute to the electrochemical performance of LNO. This behavior is accompanied by less pronounced suppression of the H1 → M → H2 → H3 phase transition in the dry‐doped samples, thereby retaining a higher discharge capacity than the wet‐doped sample.

To determine the composition of stabilized phases after synthesis of “Zr‐doped” LiNiO_2_, we have modelled and simulated a variety of possible reactions (Table 1) with density functional theory (DFT), using the projector augmented wave (PAW) method [29, 30, 31, 32] implemented in the Vienna Ab initio Simulation Package (VASP) [33]. During the temperature treatment (T>540°C) it is expected that the water of crystallization associated with hydroxides or sulphates will evaporate first [34, 35]. This is followed by formation of the metal‐oxides (Li_2_O, NiO, ZrO_2_) from the hydroxides or sulphates by further release of water and sulphur trioxide (see Table A1 in SI). Upon further heating, the metal‐oxides will react with each other to form either i) a mixture of LiNiO_2_ and Li_2_ZrO_3_ (LZO), ii) a single phase Zr‐doped LNO with Li and Ni on alkali‐metal (AM) site and/or Ni, Li and Zr on transition‐metal (TM) site, namely [Li_1_ *_‐x_* Ni*_x_*]_AM_[Ni_1_ *_‐x‐y_* Li*_x_* Zr*_y_*]_TM_O_2‐_ *_n_* or iii) [Li_1‐_ *_x_* Ni*_x_*]_AM_[Ni_1‐_ *_x‐y_* Li*_x_* Zr*_y_*]_TM_O_2‐_ *_n_* together with possible nonstoichiometric Ni‐rich phases such as Li_1‐_ *_m_* Ni_1+_ *_m_* O_2_ (see Figure 1). Similar phases have been reported near the surface region of primary particles of the Zr‐doped LiNiO_2_, where a superlattice diffraction pattern has been observed by Yoon et al. [14]. We also considered O_2_ as an additional reactant (in reactions (2), (5) & (6)), as oxygen is either present in the air or applied during synthesis [36, 37, 38, 39]. Our calculations (Figure 1a) indicate that all reactants and products listed in Table 1 are strongly exothermic (more than 1.2 eV per formula unit) compared to Li_2_O, NiO, and ZrO_2_. Hence, hereafter these simple oxides will not be discussed. The formation of Ni‐rich Li_1‐_ *_m_* Ni_1+_ *_m_* O_2_ phases, in which Ni*_m_* ions partially occupy the Li sites, is considered to form together with Zr‐doped LNO in which the same amount of Li (Li*_m_*), which has been replaced by Ni in Li_1‐_ *_m_* Ni_1+_ *_m_* O_2_, occupies Ni‐sites close to the Zr dopant. This can be seen as interchange of Li and Ni between Ni and Li layers respectively. Depending on Zr doping concentration (*y* = 1–7%) at TM sites, Zr*_y_*, the following defects were introduced to the Zr‐doped LNO system in order to keep the conservation of mass in the studied reactions: Li at the TM site Li*_x_*, Ni at the AM site Ni*_x_*, as well as vacancies at AM, TM, and/or O sites. Low amounts of such defects can occur during the synthesis depending on the experimental conditions.

**TABLE 1:** Calculated reaction energies (in meV per formula unit) for Zr‐doping of LiNiO_2_ in the absence or presence of oxygen using DFT considering fractional amounts from the models.

No	Non‐doped → Doped	*∆E* ^tot^
(1)	LiNiO_2_ + 1/96 Li_2_ZrO_3_ → 1 Li(Ni_94/96_Li_1/96_Zr_1/96_)_TM_O_2_ + 1/64 Li_2/3_Ni_4/3_O_2_	−30
(2)	LiNiO_2_ + 1/94 Li_2_ZrO_3_ + 1/188 O_2_ → 48/47 Li(Ni_94/96_Zr_1/96_)_TM_O_2_	−29
(3)	LiNiO_2_ + 1/48 Li_2_ZrO_3_ → 1 Li(Ni_92/96_Li_2/96_Zr_2/96_)_TM_O_2_ + 1/32 Li_2/3_Ni_4/3_O_2_	−17
(4)	LiNiO_2_ + 1/32 Li_2_ZrO_3_ → 1 Li(Ni_90/96_Li_3/96_Zr_3/96_)_TM_O_2_ + 3/64 Li_2/3_Ni_4/3_O_2_	−5
(5)	LiNiO_2_ + 3/91 Li_2_ZrO_3_ + 1/182 O_2_ → 96/91 Li(Ni_91/96_Li_1/96_Zr_3/96_)_TM_O_2_	−20
(6)	LiNiO_2_ + 1/25 Li_2_ZrO_3_ + 1/50 O_2_ → 27/25 Li_26/27_(Ni_25/27_Li_1/27_Zr_1/27_)_TM_O_2_	−30
(7)	LiNiO_2_ + 1/25 Li_2_ZrO_3_ → 27/25 Li_26/27_Ni_1/27_(Ni_24/27_Li_1/27_Zr_1/27_)_TM_O_53/27_	+66
(8)	LiNiO_2_ + 1/25 Li_2_ZrO_3_ → 27/25 Li(Ni_25/27_Zr_1/27_)_TM_O_53/27_	+57
(9)	LiNiO_2_ + 1/25 Li_2_ZrO_3_ → 27/25 Li_26/27_(Ni_25/27_Li_1/27_Zr_1/27_)_TM_O_53/27_	+27
(10)	LiNiO_2_ + 1/12 Li_2_ZrO_3_ → 9/8 Li(Ni_24/27_Li_1/27_Zr_2/27_)_TM_O_2_	+34

**FIGURE 1:**
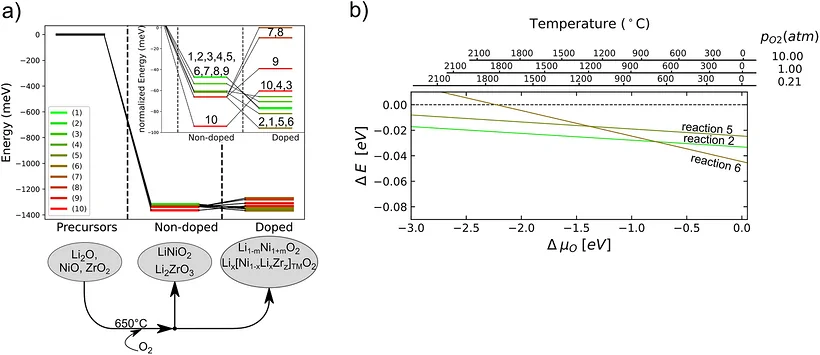
a) Visualized reaction energies of doped and non‐doped cases from Table 1 relative to the synthesis precursors. A closer view (inset) shows the reaction energies with respect to the highest energy value (66 meV from reac. (7), schematic illustration below showing possible intermediate and final reactions during Zr‐doping of LNO); b) Computed energies of reactions 2, 5 and 6 as function of chemical potential of oxygen (μ_O_), referenced to $E_{O_{2}}^{\mathrm{tot}}$ using ab‐initio atomistic thermodynamics approach. The temperature dependence for three likely oxygen partial pressures is presented on the top.

Our calculations (Table 1) show that the reactions between Li_2_ZrO_3_ and LiNiO_2_ can lead to [Li*_1‐x_* Ni*_x_*]_AM_[Ni*_1‐x‐y_* Li*_x_* Zr*_y_*]_TM_O_2‐_ *_n_* with low concentrations of Zr lattice doping of 1/96 (∼1%) to 3/96 (∼3%) (in reactions (1)—(5)). Zr doping can be achieved with and without oxygen. However, the energy preference becomes significantly larger with O_2_ in case of ∼2% (2/96) or 3% of Zr doping. Without oxygen, a small amount of second phases of Li_1‐_ *_m_* Ni_1+_ *_m_* O_z_ such as Li_2/3_Ni_4/3_O_2_, whose concentration is higher for larger amount of Zr‐concentration, forms besides Zr‐doped LNO (in reactions ((1), (3) & (4)). Zr‐doped LNO and second phase Li_1‐_ *_m_* Ni_1+_ *_m_* O_z_ can be viewed as one phase with interchange of Li and Ni between Li and Ni sites. This is because Zr^4+^ can be stabilized at TM site only with Li in its vicinities. This reduces the strong electrostatic repulsive interaction between Zr^4+^ and its nearest neighbor ions (charge analysis is shown in Figure 7).

For reactions (5) and (6), additional Li occupying TM sites was taken into account, and for all three reactions (2), (5) and (6), vacancies at Li and TM sites were applied in order to form Zr‐doped LNO, namely Li[Ni*_1‐x‐y_* Li*_x_* Zr*_y_*]_TM_O_2_. These reactions are found to be energetically preferred by ‐29 meV (1% Zr), ‐20 meV (3% Zr) and ‐30 meV (4% Zr), respectively. In general, the increased Zr dopant concentration leads to higher reaction energies, which indicates a decreased energetic preference toward stable bulk doping with Zr for high Zr doping concentration. For 3% Zr doping, the energy preference is very small, but with oxygen it became larger. In case of ∼4% (1/27) of Zr doping, this is only possible with applying oxygen as reactant.

To maintain the charge neutrality and mass conservation at increasing Zr‐dopant content (4% to 7%), vacancies at Li, O and TM sites are introduced (reactions (7)‐(9)), as well as the addition of Li to TM sites (reactions (7), (9), and (10)), namely Li*_z_* [Ni*_1‐x‐y_* Li*_x_* Zr*_y_*]_TM_O_2‐_ *_n_* with *z*≤1, and *n*>0. Most of these reactions with 4% Zr content are unfavorable: reaction (7): *ΔΕ^tot^* = +66 meV, reaction (8): *ΔΕ^tot^* = +56 meV, and reaction (9): *ΔΕ^tot^* = +27 meV. Only in reaction (6), Li_2_ZrO_3_ reacts with LiNiO_2_ in contact with O_2_ leading to a relatively large amount of Zr doping of 4%. In this case, no second phase forms and the reaction energy is *ΔΕ^tot^* = ‐30 meV. Large amount of Zr lattice doping, such as ∼7% (2/27) (reaction (10)) is energetically very unfavorable to produce a single phase [Li*_1‐x_* Ni*_x_*]_AM_[Ni*_1‐x‐y_* Li*_x_* Zr*_y_*]_TM_O_2‐_ *_n_* with *ΔΕ^tot^* = +34 meV.

Furthermore, we computed the formation energy of reactions (2), (5) and (6) as function of temperature (*T*) and partial pressure of O_2_ (*p*_O2_) (details provided in SI). Figure 1b illustrates the (corrected) energies for reactions (2), (5) and (6) as function of the chemical potential of oxygen $\Delta \mu_{O}\left(T,p_{O_{2}}\right)$ as well as temperature for different oxygen partial pressures $p_{O_{2}}$. Since the $p_{O_{2}}$ value cannot easily be quantified during the synthesis, we applied the ambient pressure of $p_{O_{2}}$= 0.21 atm as well as 1 atm and 10 atm (∼1 MPa), the last one represents a relatively oxygen‐rich condition. The computed energies of reactions (2), (5) and (6) as function of temperature and pressure with the aforementioned corrections indicate a linear decrease in absolute values of Δ*E*(μ_O_) as the chemical potential of oxygen decreases independent of Zr dopant concentration. It is found that at room temperature (20‐30°C) the forward reaction (6) is still energetically favorable in which the doping of LiNiO_2_ bulk with Zr is achieved by bringing LiNiO_2_ in contact with Li_2_ZrO_3_ (or LiO and ZrO) and oxygen. This result is not much dependent on the applied partial pressures of O_2_ gas. However, by increasing the temperature, i.e. calcination, the energy preference of reactions (2), (5) and (6) decrease. LNO with Zr doping of equal and less 3.7% is energetically favorable in the air and up to 10 atm for all reasonable temperatures.

The chemical compositions of the 1%, 3%, and 7% Zr dry‐doped cathode materials closely match the target values, as confirmed by ICP‐OES analysis (Table A3 in SI). However, such compositional consistency does not necessarily indicate the successful incorporation of Zr into the cathode crystal structure. Because ICP‐OES involves dissolving the entire cathode material through acid treatment, the obtained results reflect the overall bulk composition rather than the composition of the cathode active materials. Figure 2a presents the full XRD pattern of the respective cathode materials, with magnified views. These data reveal that Zr is not fully incorporated into the cathode crystal structure. The XRD pattern exhibits the conventional hexagonal layered structure belonging to the $R\bar{3}m$ space group (Figure 2a). However, close inspection shows the formation of Li_2_ZrO_3_, of which the intensity increases with higher Zr doping amounts (see Figure 2a). This implies that a portion of the Zr reacts with LiOH during calcination, leading to the formation of Li_2_ZrO_3_ and its segregation outside the primary particles. A successful doping of only very small amount of Zr to LNO can be further confirmed by comparing theoretical and experimental lattice parameters. For this purpose, the *a* and *c* lattice parameters are displayed in Figure 2b for undoped and 1% – 7% Zr‐doped LNO from theoretical and experimental case. Theoretically determined lattice parameters of Zr‐doped LNO are significantly larger compared to those obtained from XRD measurements (Figure 2b and Figures A3, A4 in SI). This indicates that only a fraction of Zr occupies the lattice sites, and the rest of dopant species probably segregates to the surface of LNO particles and/or form Li_2_ZrO_3_ impurities (in the case of 3 and 7% Zr, see Figure 2a).

**FIGURE 2:**
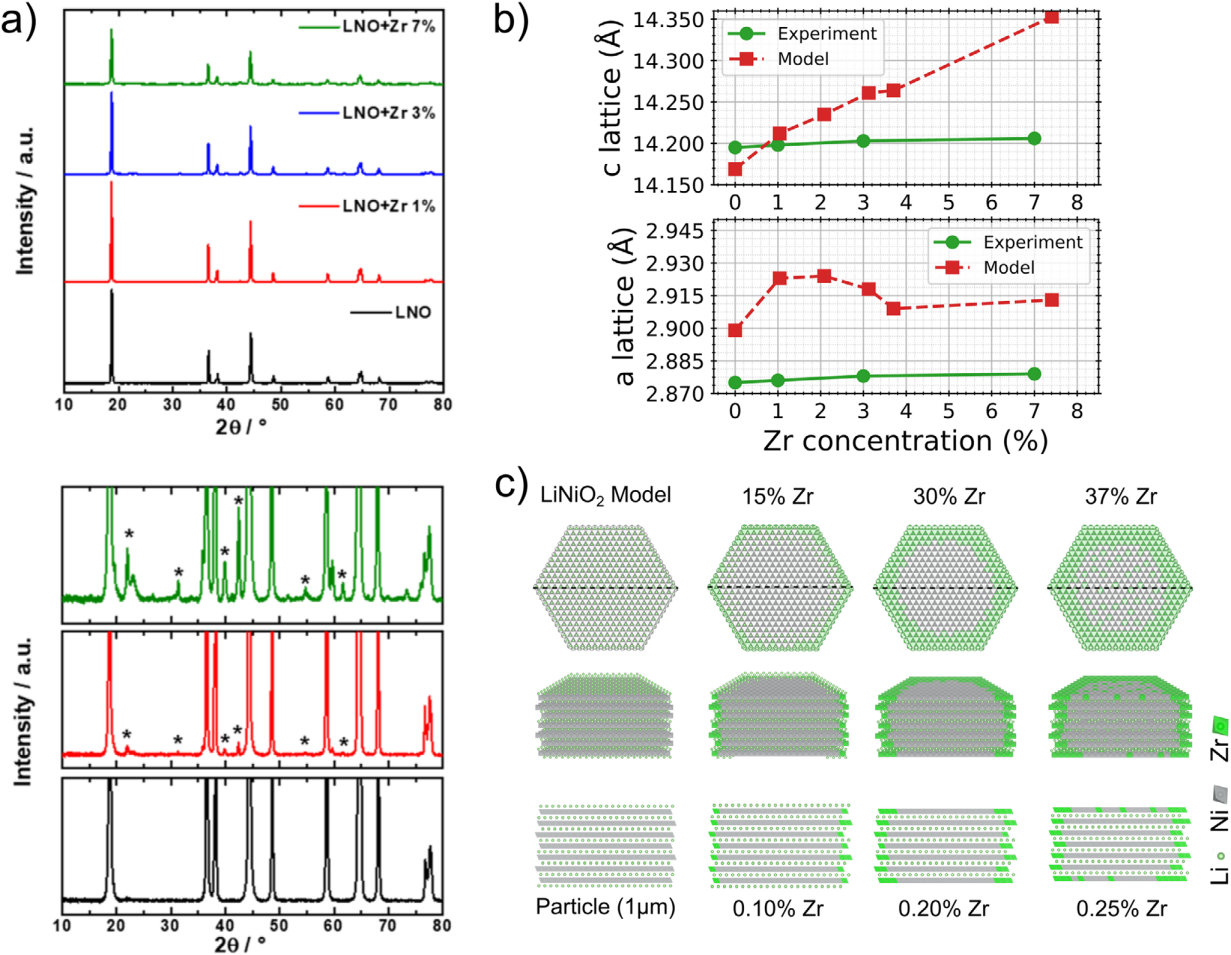
a) Experimental XRD patterns of LNO and Zr‐doped LNO samples prepared by the dry doping method (top), and magnified XRD patterns highlighting the secondary phases (bottom). The asterisks (*) indicate the characteristic peaks of Li_2_ZrO_3_; b) experimental and DFT‐calculated *a* and *c* lattice parameter as function of Zr‐concentration; c) top‐, 3D and side views of simulated (using electrostatics and REMC) LNO particles doped with different Zr‐concentrations.

To confirm the segregation of Zr to the surface of cathode grains, we modelled LNO particles with a large atomistic structure containing about 12,000 atoms (Li_3130_ Ni_2710_ O_6096_) where different Zr concentrations of 15%, 30% and 37% were introduced. By optimizing configurational atomistic structure of particle using electrostatic analysis (Coulomb energies) and Replica Exchange Monte Carlo (REMC) with the global optimization of atomistic configurations by Coulomb (GOAC) code [53], we found that Zr ions prefer to occupy at first the outermost Ni layers at the lateral surface and then the base surface of particles. The crosscut of these particle models (Figure A5 in SI) shows, that no Zr is present in the bulk region of the particle. The ratios of Zr/Ni (concentration of Zr doping) that we have studied in our models are 15%, 30% and 37% presented in Figure 2c. Since our calculations show that the majority of Zr ions occupies the surface of particles, we can estimate the concentration of Zr at the surface of primary particles in Figure 3. The concentration of Zr (surface) doping is estimated (details provided in section A1 in SI) to be between 1.24% and 2.48% for typical particle sizes between 200 nm and 100 nm (see Figure 3). As the refined lattice parameters by XRD (Figure 2b) are significantly smaller than our calculated values for LNO with Zr doping of 1% to 7% we conclude that Zr preferably substitute surface Ni sites and only a very small fraction of Ni in the bulk is replaced by Zr. Since the computed formation energy for Zr doping decreases with dopant concentration, we can conclude that large amounts of Zr doping to the lattice is unlikely. However, as Table 1 shows, the absolute value of the reaction energy at low concentration of Zr is very large (reaction 2 and 5) and is expected to be much larger for concentrations below 1% percentage. This might mean, after the surface of particle is fully covered by Zr, a small amount of Zr doping to the lattice can be realized. However, increasing of Zr concentration (reaction 10) leads to the formation of Li_2_ZrO_3_ impurity.

**FIGURE 3:**
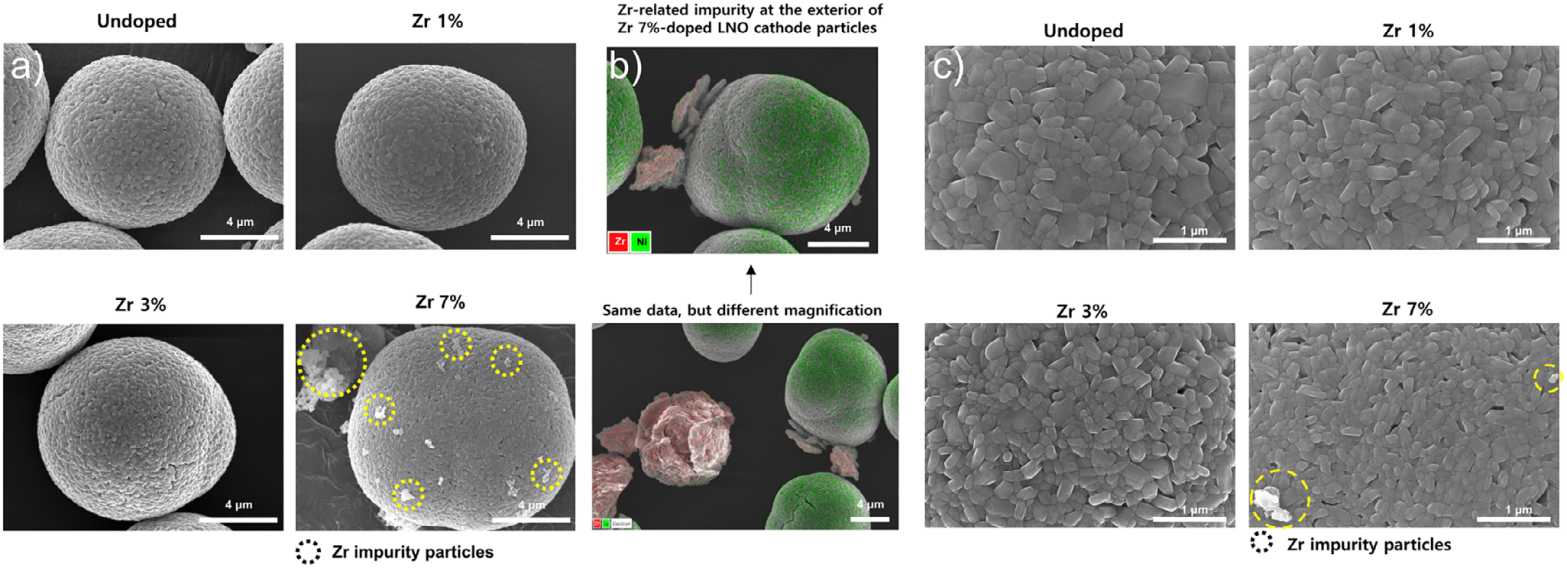
a) SEM and b) SEM‐EDS results of the undoped and (1%–7%) Zr‐doped LNO cathode samples prepared by the dry doping method. c) High‐magnification SEM images of the cathode materials. Yellow marked circles indicating impurities.

The morphology and size of the particles for dry‐synthesized LNO particles, namely undoped LiNiO_2_ and doped with 1%, 3% and 7% of Zr, can be observed in Figure 3 using SEM and SEM‐EDS as well as HR‐SEM methods. The aforementioned particles in the respective SEM images (Figure 3a) are all spherical shaped with a diameter of ∼8 – 11 µm and an excess of Zr‐doping (7%) exhibits additional impurity particles outside the cathode grains. SEM‐EDS results (Figure 3b) show that these impurities are predominantly composed of Zr, confirming the segregation of Zr near the surface of the cathode grains forming on‐surface Zr doping (Figure 2) and/or formation of Li_2_ZrO_3_ (Table 1 and Figure 1) as both have been predicted by our simulation and measured by the XRD results (Figure 2a) for 3% and 7% Zr‐doped LNO. Upon further magnification by HR‐SEM images (Figure 3c), it can be seen that size of the primary particles on the surface changes from ∼225 nm (undoped LiNiO_2_) to ∼90 nm (7% Zr‐doped) as well as their morphology appear rod‐shaped in case of Zr‐doped LNO. The reduction in grain size suggests that an additional size‐controlling factor contributed during the calcination of cathode material, most likely the segregation of Zr along the cathode surface and grain boundaries as was predicted by simulation (Figure 2).

Cross‐sectional SEM images (Figure 4) of the synthesized cathode materials show that the decrease in primary particle size at high Zr concentration is not only on the exterior, as previously observed in Figure 3, but also in the interior of the secondary particle. In case of the Zr‐doped LNO, the primary particles on the exterior are more elongated. The computed lattice strains (Δ*a*, Δ*c*) in secondary phases, namely Li_2/3_Ni_4/3_O_2_, with respect to LiNiO_2_ are $\Delta a=\left(a_{Li_{2/3}Ni_{4/3}O_{2}}--a_{\mathrm{LiNi}O_{2}}\right)/a_{\mathrm{LiNi}O_{2}}$ = 1.97% and $\Delta c=\left(c_{Li_{2/3}Ni_{4/3}O_{2}}--c_{\mathrm{LiNi}O_{2}}\right)/\;c_{\mathrm{LiNi}O_{2}}=0.83\%$. This might demonstrate the tendency for the formation of Li_2/3_Ni_4/3_O_2_ particles radially on the surface of secondary particles (Figure 4). The particles with *a*‐axis along the radial direction on the surface of aggregate which contain cation ordering (so‐called second phases in reactions 1, 3, and 4) can accommodate the arising large strain along *a*‐direction due to their formation. Simultaneously, Zr dopants occupy mainly the surface Ni sites and only low amounts of Zr on the bulk Ni sites with Li in their vicinity. The replaced Ni ions by Zr and occupying Li ions around Zr at TM sites in the Zr‐doped LNO should occur simultaneously with an excess of Ni and a lack of Li as in the case in Li_2/3_Ni_4/3_O_2_.

**FIGURE 4:**
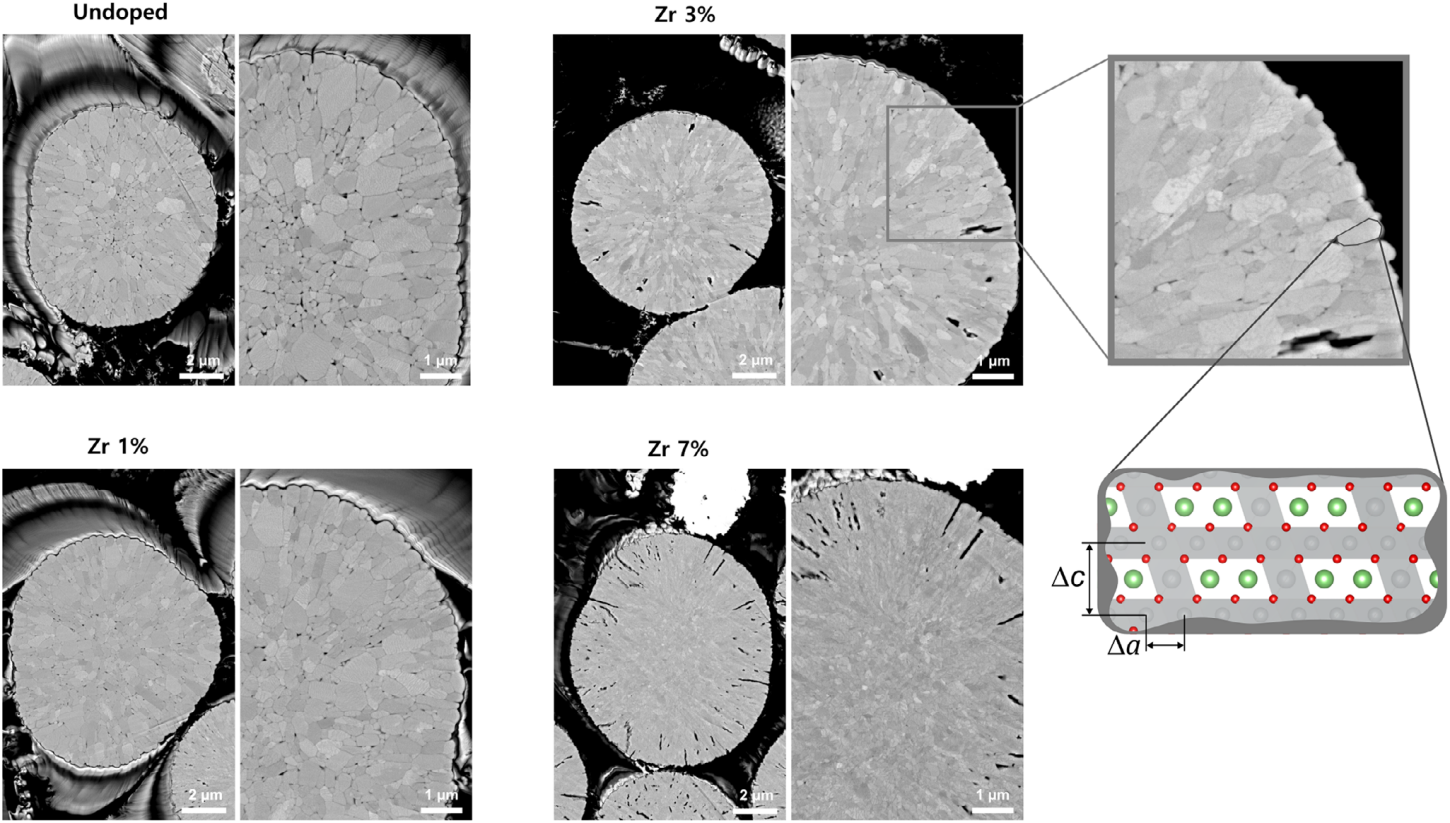
Cross‐sectional SEM results for undoped and (1%–7%) Zr‐doped LNO particles prepared by the dry doping method (left); DFT lattice strains in Ni‐rich second phases with respect to non‐doped LNO (right).

Figure 5 presents TEM results of a Zr 3%‐doped LNO cathode particle. The bright‐field STEM image shows that the peripheral grains are radially oriented and elongated, whereas the central grains are relatively short and equiaxed (Figure 5a). Two main factors are responsible for the radial arrangement and elongation of primary particles: physical effects and crystallographic considerations. Given that the morphology of the Ni(OH)_2_ precursor resembles that of the Zr 7%‐doped LNO, Zr plays a role in maintaining the grain morphology of precursor. From a physical view point, the segregation of Zr residues along the grain boundaries of the cathode material likely hampers the consolidation of constituent grains, i.e. sintering, during calcination, thereby preserving the precursor grain morphology [40]. This morphology represents a strategy opposite to the approach of enlarging grain size to produce single particles for enhancing the performance of Ni‐rich cathodes [41]. Instead, it corresponds to an alternative approach in which the grains of polycrystalline cathodes are nano‐sized to form nano‐rod grains, thereby decreasing anisotropic straining and improving cycling performance. The corresponding SAED patterns obtained from these grains differ markedly. The SAED pattern of the central grain in Figure 5b displays electron diffraction spots characteristic of the conventional layered structure. In contrast, the SAED pattern of the peripheral grain Figure 5c reveals the formation of superstructure, characterized by regular intermixing of TM and Li^+^ cations along the TM and Li layer, indicating the formation of cation‐ordered structure [42]. TEM and SAED results of numerous grains presented in the Supplementary Information provide further evidence supporting these observations (Figure A6).

**FIGURE 5:**
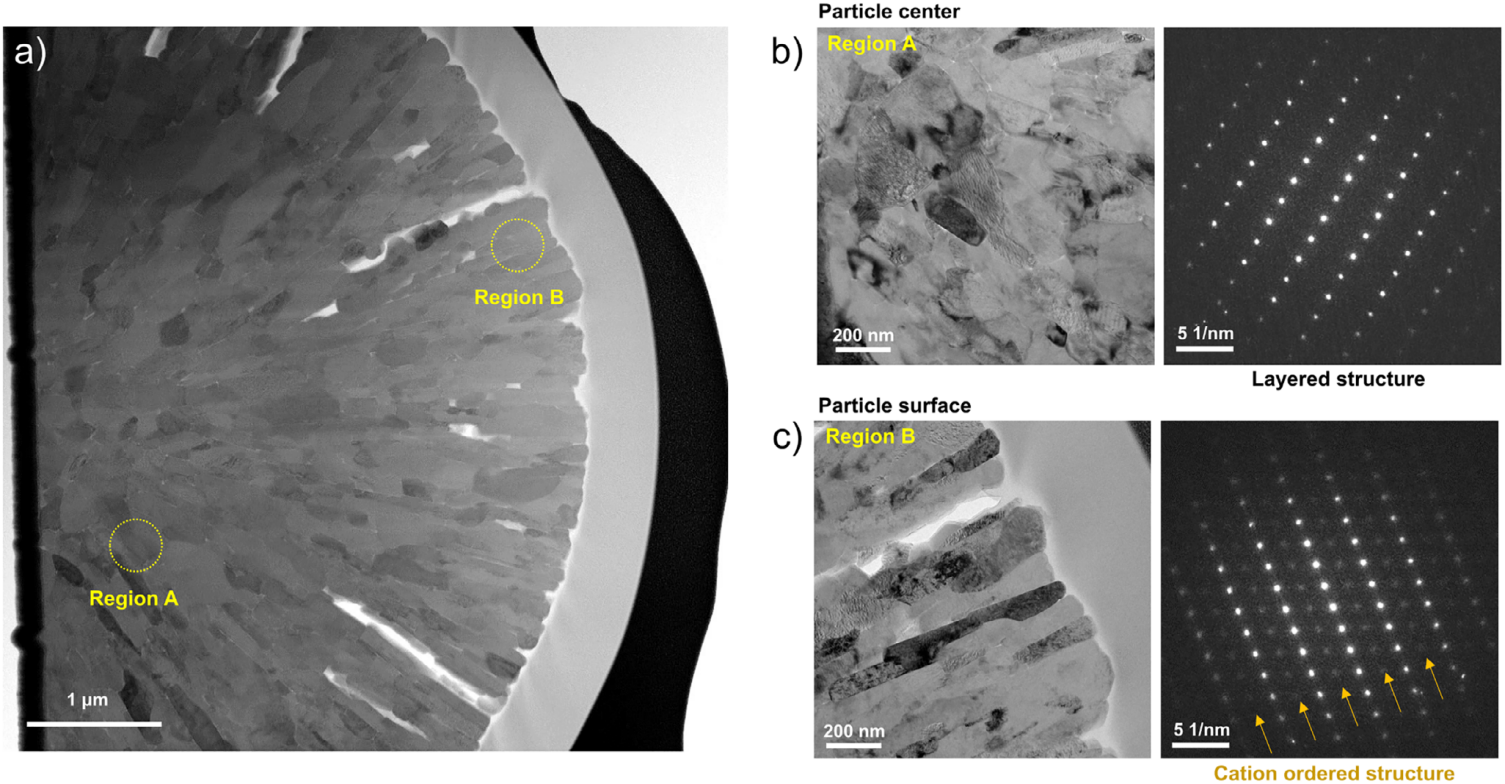
Crystal structure by cross‐sectional STEM (a)) and TEM of Zr 3%‐doped LNO sample prepared by the dry doping method: Region A (in b)) and of region B (in c)), yellow marked Region B showing cation‐ordered superstructure imposed on the typical layered structure.

Simulated SAED pattern in the [100], [110] and [001] zone were generated (Figure 6) for the following LNO models: pristine LiNiO_2_, Ni‐rich Li_1‐x_Ni_1+x_O_2_, Ni‐poor LiNi_1‐x_O_2_ and LiNiO_2_ with Li/Ni‐interchange [Li_1+x_Ni_x_]_AM_[Ni_1‐x_Li_x_]_TM_O_2_, each with a substitution of x = 0.25. In the Ni‐rich case, the structure was built by inserting Ni ions on Li sites in an ordered configuration (See Figure A8 in SI). In comparison with the experimental SAED patterns in Figure 5, it can be observed that the pattern of pristine LNO in [100] and [110] zones match best with the SAED pattern for grains in the interior (region A). The simulated SAED pattern of Ni‐rich LNO in [110] zone shows the same pattern (Figure 6, yellow arrows) as the experimental SAED pattern of the exterior of secondary particles (Figure 5, region B). This confirms the formation of Ni‐rich structures which has been predicted (Table 1 and Figure 1) as second phases in coexistence with the Zr‐doped LNO at low Zr concentrations (<4%). The simulated SAED pattern of the other structures, namely Ni‐poor LNO (LiNi_1‐x_O_2_) and LNO with Li/Ni interchange ([Li_1+x_Ni_x_]_AM_[Ni_1‐x_Li_x_]_TM_O_2_), is different from the experimental SAED pattern in Figure 5, which ensures that the composition of the particles close to the exterior neither contains Ni vacancies nor involves the sole Ni/Li interchange (without Ni enrichment).

**FIGURE 6:**
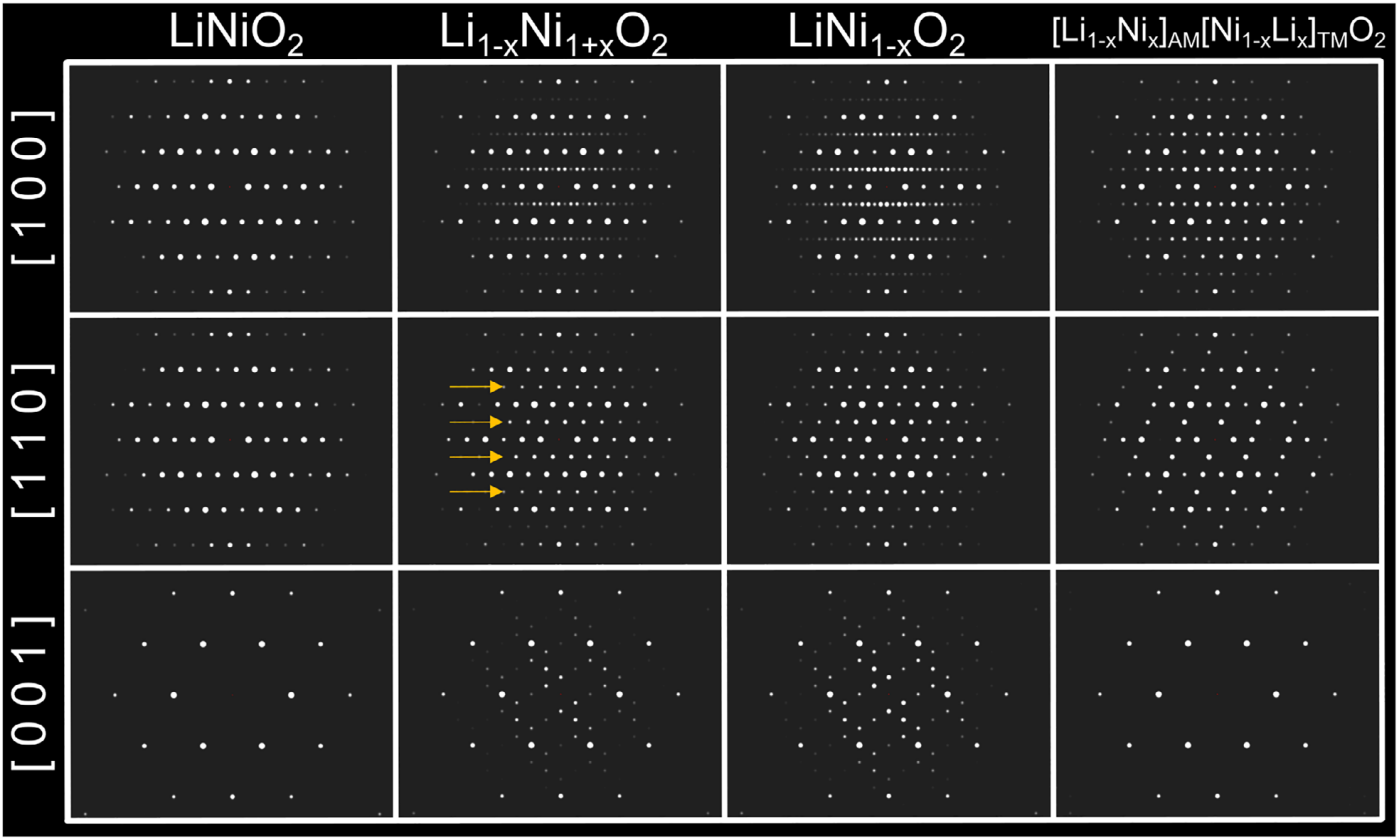
Simulated electron diffraction pattern for different possible LNO structures at different zones. The orange arrows indicate the cation ordered region observed in experimental SAED (Figure 5c).

The electrochemical performance of the cathode materials was evaluated via half‐cells. Figure 7a presents charge–discharge curves of the respective half‐cells at 0.1 C and 2.7–4.3 V. The discharge capacities of the pristine, 1%, and 3% Zr‐doped LNO cathodes are 251, 249, and 244 mAh/g, respectively, reflecting the decreased average Ni content in the Zr‐doped cathodes. However, the 7% Zr‐doped LNO exhibits a markedly lower initial charge and discharge capacity of 228 and 207 mAh/g, respectively, attributed to the presence of substantial Zr‐associated impurities (Li_2_ZrO_3_, as predicted in Table 1 and observed in Figure 2a) that increases the resistance of the cathode. The initial Coulombic efficiency remains as high as 97% up to 3% Zr doping, but it decreased significantly to 91% in the Zr 7%‐doped LNO. The cycling performance of the cathodes at 0.5 C is presented in Figure 7b. The undoped LiNiO_2_ retained 73% of its initial capacity after 100 cycles, whereas the addition of 1% Zr greatly enhanced cycling stability, achieving 80% capacity retention. The 3% Zr‐doped LNO exhibited the best cycling performance, delivering 84% of capacity retention without substantial compromise in initial capacity. The 7% Zr‐doped LNO showed the highest capacity retention (93%), however, this was accompanied by a pronounced decrease in initial capacity. It is well established that Li^+^ insertion and extraction in Ni‐rich cathodes induce severe anisotropic strain, which deteriorates structural stability and ultimately degrades the cycling performance of the cathode [4, 43]. The radially oriented rod‐shaped primary particles in Zr‐doped LNO may help to alleviate accumulation of anisotropic strain, thereby enhancing the cycling stability of the cathode [44]. The regularly ordered TM ions in the Li layers of the cation‐ordered structure may help to maintain structural robustness, even during Li^+^ extraction from the host structure [14, 17]. Zr dopants at the surface of secondary particles can protect the cathode from aggressive HF reaction from electrolyte [14, 45]. In addition, Zr at the grain boundary can help to maintain the integrity of macrostructures during charge/discharge [17]. For comparison, it is reported that LiNiO_2_ has a high theoretical capacity of 274 mAh/g, and experiments observed first charge capacity of up to 245 mAh/g at a 0.1 C rate tested between 2.7 to 4.3 V at room temperature, but showing a strong irreversible capacity loss, as only a Coulombic efficiency of 74% has been observed after 100 cycles at 0.5 C rate [46, 47]. Recent studies demonstrated, that the incorporation of elements with small ionic radius or high charges, such as B^3+^, Mo^4+,6+^, and W^6+^ into LiNiO_2_ can modify the structural stability in a way that it enhances the capacity retention significantly. For example, Boron doping into NCA, namely LiNi_0.878_Co_0.097_Al_0.015_B_0.01_O_2_ leads to an increased formation of rod‐shaped primary particles in the outer region of the secondary particles. This seems to stabilize the microstructure integrity, resulting to an improved capacity retention of 93.5% under similar conditions [24]. In case of Mo doping into a Li‐rich Ni‐rich cathode (Li_1+y_Ni_(3‐5y)_Mo_2y/3_O_2_ with y from 0.03 up to 0.12), a phase separation was observed with two domains, one Mo‐rich (such as Li4MoO5) and one LiNiO2‐rich, which appears to stabilize the capacity up to 91% [48]. Doping of Ni‐rich cathodes with W has been found to be unlikely as W prefers to kind of form coating layers rather than occupying the lattice sites. The resulting Li_x_W_y_O_z_ phases enhance the capacity retention of the cathode material [36]. These works are in line with our study showing a small amount of doping with an appropriate element can enhance the cyclability of Ni‐rich cathodes by modifying surfaces, grain boundaries and/or interfaces.

**FIGURE 7:**
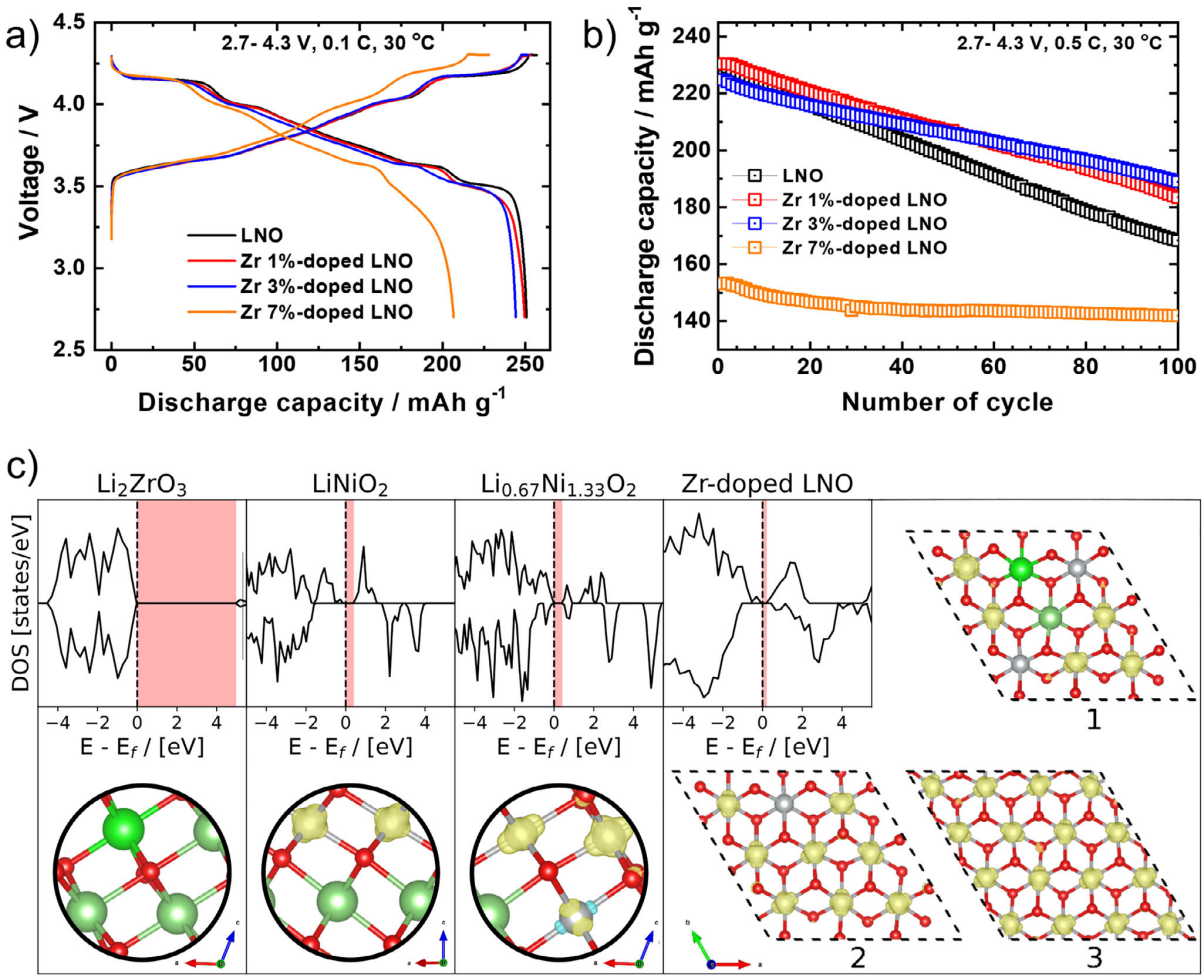
a) Charge–discharge voltage as function of capacity curves and b) cycling performances of undoped LiNiO_2_ and (1%–7%) Zr‐doped LNO cathodes in half‐cell. c) Hybrid DFT/HF computed density of states (DOS) and spin density difference (SDD) for reactants (non‐doped) and selected products (doped) from reaction (6) in Table 1.

The density of state (DOS), band gap (BG), as well as the spin density difference (SDD) were computed (see Figure 7c) using DFT‐HSE calculation for LiNiO_2_, Li_2/3_Ni_4/3_O_2_, LZO, and a ∼4% (1/27) Zr‐doped LNO, namely Li_26/27_(Ni_25/27_Li_1/27_Zr_1/27_)_TM_O_2_ (reaction (6) as an example from Table 1). For the last case, a low concentration of ∼4% was selected to meet the computational demand of HSE calculation, but is still comparable to the experimental values (see details in SI p.1). In the case of pristine LiNiO_2_, there is a BG of 0.38 eV, which is close to the experimental value of 0.5 eV reported by Molenda et al. [49]. Computed BG for Li_2/3_Ni_4/3_O_2_ is 0.38 eV, similar to that of LiNiO_2_. Nevertheless, calculated BG of LZO is 5.19 eV (reported experimental values are 5.43 to 5.50 eV [50]) which is much larger than the LNO cases. This indicates the insulating character of the Zr impurities, which might be one reason for the reduced initial capacity. Other reasons can be an increased Li/Ni mixing or the release of oxygen. The lowest BG is obtained for the Zr‐doped structure, namely Li_26/27_(Ni_25/27_Li_1/27_Zr_1/27_)_TM_O_2,_ which is 0.18 eV. This shows that a low concentration of Zr^4+^ can have a positive effect on the overall conductivity, which might be due to the formation of Ni^2+^ by replacing Ni^3+^ by Zr^4+^ to maintain the charge neutrality of the system. Due to the replacement of TM by Li ions, the presence of Ni^4+^ is expected as well (to maintain charge neutrality). Calculated magnetic moments confirm the presence of 4% Ni^2+^,78% Ni^3+^, 11% Ni^4+^, and 4% of Zr^4+^, which can enhance electronic conductivity in terms of small polaron hopping between Ni of different charge states. The computed shape of spin density difference (SDD) from Figure 7 also visualizes disproportion of charges on cations. In case of LiNiO_2_, all Ni ions show the characteristic spin shape of Ni^3+^ ([Ar]3d^7^, t_2g_^6^ e_g_^1^), which is plausible due to conservation of charge. For the Ni‐rich composition (Li_2/3_Ni_4/3_O_2_) alternating spin shapes of Ni^3+^ and Ni^2+^ ([Ar]3d^8^, t_2g_^6^ e_g_^2^) are observed on Ni sites and Ni^4+^ ions ([Ar]3d^6^, t_2g_^6^ e_g_^0^ i.e. no SDD) can be seen together with Li^+^ ions on Li sites. In case of LZO, no spin density is visible, due to the presence of Zr^4+^ ([Kr], t_2g_^0^ e_g_^0^, i.e. no SDD). For the case of Zr‐doping, the disproportion of charges on Ni ions (coexistence of Ni^2+^, Ni^3+^, and Ni^4+^) can be clearly observed from the corresponding SDD.

## 3 Conclusion

Our electrostatic analysis and first‐principles calculation predict that up to 3–4% Zr can be incorporated into the LiNiO_2_ lattice, provided that either a minimal amount of Ni‐rich secondary phases (Li_1‐_ *_m_* Ni_1+_ *_m_* O_2_) form or an oxygen‐rich environment is maintained during calcination. When 3% of Zr‐doping is used, a cation‐ordered superlattice (in Li_1‐_ *_m_* Ni_1+_ *_m_* O_2_) is observed in SAED patterns, which was further confirmed by simulated diffraction patterns. Ab initio atomistic thermodynamics calculation validates that the oxygen incorporation into LiNiO_2_ remains favorable during Zr doping even at elevated temperatures. Our large‐scale atomistic simulations of LiNiO_2_ particles using electrostatic analysis and Replica Exchange Monte Carlo (REMC) optimizer predict that Zr preferentially occupies surface Ni sites. Moreover, Zr doping promotes the formation of radially elongated primary particles due to the sintering retardant effect of surface‐segregated Zr and the development of a secondary structure. These findings were experimentally corroborated by XRD, cross‐sectional SEM, and SAED and showed good agreement with DFT calculation results. When Zr is added in excess, it tends to form Li_2_ZrO_3_ impurities instead of being incorporated into the crystal lattice, and this excessive impurity formation can reduce the reversible capacity, indicating that only a doping level sufficient to induce (micro)structural modification is desirable. Overall, the combination of electrostatic, REMC, DFT, and thermodynamic techniques predicts complex doping behavior, which aligns well with experimental results, and the identified Zr doping mechanism demonstrates that (micro)structural engineering of LiNiO_2_ cathodes through controlled Zr incorporation can significantly enhance battery cycling performance.

## Author Contributions

L.W.: methodology, investigation, validation, visualisation, writing – original draft & editing.G.‐T. P.: investigation, validation, visualisation, writing – original draft & editing.L.‐Y. K.: conceptualisation, methodology, investigation, writing – original draft. M.I.: conceptualisation, methodology, investigation, writing – original draft. K.K.: validation, visualisation, writing – review & editing. J.E.E.: methodology, supervision, writing – review & editing. M.H.: methodology, supervision, writing – review & editing. K.: Investigation, Methodology. M.‐G. S.: Investigation, Methodology. Y.‐K. S.: methodology, supervision, writing – review & editing. P.K.: methodology, supervision, writing – review & editing.

## Conflicts of Interest

The authors declare no conflicts of interest.

## Supporting information




**Supporting File**: advs78453‐sup‐0001‐SuppMat.docx.

## Data Availability

The data that supports the findings of this study are available in the supplementary material of this article.
