# Supplementary material for: Zr Incorporation Into Lithium Nickel Oxides: Bulk or Surface Substitution?
Source: Adv Sci (Weinh). 2026 Nov 4:e78453. Online ahead of print. doi: 10.1002/advs.78453 (PMC13736196; doi:10.1002/advs.78453)
Supplement: Supplementary file 1 [file ADVS-9999-e78453-s001.docx]

**Supporting information of**

**“Zr Incorporation into Lithium Nickel Oxides: Solid Solution or Two-Phase System?”**

Lars Winkler^[1]+[a,e]^, Geon-Tae Park^+[b]^, Liang-Yin Kuo^[c]^, Martin Ihrig^[2]^Δ^[d]^, Konstantin Köster^[a,e]^, Johan E. ten Elshof^[e]^, Mark Huijben^[e]^, Myoung-Chan Kim^[b]^, Min-Gyu Seo^[b]^, Yang-Kook Sun^[3]*[b]^, and Payam Kaghazchi^*[a,e]^

^[a]^Institute of Energy Materials and Devices (IMD) – IMD-2: Materials Synthesis and Processing, Forschungszentrum Jülich GmbH, 52425 Jülich, Germany

^[b]^Department of Energy Engineering, Hanyang University, Seoul 04763, South Korea

^[c]^Chair for Theoretical Physics VII and Bavarian Center for Battery Technologies, University of Bayreuth, Universitaetsstr. 30, 95447, Bayreuth, Germany

^[d]^Department of Chemical Engineering, National Taiwan University of Science and Technology, No. 43, Keelung Rd., Sec. 4, Da’an Dist. Taipei City 106, Taiwan

^[e]^MESA+ Institute for Nanotechnology, University of Twente, Enschede 7500AE, The Netherlands

**1. Computational methods**

Spin-polarized DFT calculations were performed using projector augmented wave (PAW) [1] method implemented in the Vienna Ab initio Simulation Package (VASP) code [2]. The geometries were optimized with the Perdew-Burke-Enzerhof (PBE) [3] exchange-correlation energy. A cut-off energy of 520 eV and an electronic convergence criterion of 10^−4^ eV as well as a force convergence criterion of 2x10^−2^ eV Å^−1^ were used. Density of states (DOS) and spin density difference (SDD) were calculated with the HSE06 hybrid functional [4,5] as a single point calculation with the same convergence criterion and cut-off energy as geometry setup. The precursors (Li_2_O, NiO, ZrO_2_), undoped and Zr-doped lithium nickel oxides (according to the compositions in **Tab. 1**) as well as Li_2_ZrO_3_ and Li_1-m_Ni_1+m_O_2_ were modelled using listed settings in **Table A4** and **Table A5**. The O_2_ molecule was placed in an a,b,c lattice space of 22x23x24 Å with 1x1x1 k-point mesh to prevent unphysical behavior. Regarding the DFT-HSE calculation of Zr-doped LNO, models with reasonable sizes (containing 108 atoms) can be considered due to the increased computational demand, for which we chose the lowest possible Zr doping concentration, which is approximately 4% (1/27). Lower Zr concentration, such as 3% or 1% require substantially larger models with 384 atoms (see **Tab. A4** in SI). Therefore, the 4% Zr model was chosen as a practical compromise between computational feasibility and relevance to experimental value of 3% Zr.

Coulomb energy (electrostatic analysis) was calculated with the global optimization of atomistic configurations by Coulomb (GOAC) code [6]. Compositions with less than 10^13^ combinations were solved exactly using the Gurobi solver [7], and for compositions with more combinations, the heuristic replica-exchange Monte-Carlo (REMC) method within GOAC [6] was used. To maintain charge balance, charges of O^2-^, Li^+^_,_ and a variable charge of Ni^X+^ were applied for cations and anions in the bulk models. In the particle model, charge stares of Ni^3.x+^, Li^+^_,_ and a variable charge of oxygen O^2.x-^ were defined to maintain charge balance, when 15%, 30% and 37% of Zr^4+^ is introduced on TM sites. No periodicity was consideration for Coulomb energy of calculation of particles. Electronic investigations were performed for the models listed in **Table A5**.

Simulated SAED patterns for LNO structures (2x2x1 unit cell), as pristine (LiNiO_2_), Ni-rich (Li_1-x_Ni_1+x_O_2_), Ni-poor (LiNi_1-x_O_2_) and with Li/Ni-interchange ([Li_1+x_Ni_x_]_AM_[Ni_1-x_Li_x_]_TM_O_2_) with x= 0.25 were generated, based on Bloch-wave solution of Bethe’s dynamical diffraction equations, with reflection positions determined from the Laue condition and Ewald-sphere construction within the ReciPro software by Seto et al. [8].

The atomistic structures and SDD (with iso surface value of 0.05) were visualized using visualization for electronic and structural analysis (VESTA) [9].

The formation energy of reactions (2), (5) and (6) can be calculated as function of temperature (*T*) and partial pressure of O_2_ (*p*_O2_) as follows:

$\Delta E\left( \mu_{O} \right)=\Delta E^{\mathrm{tot}}-c\Delta\mu_{O_{2}}\left( T,p_{O_{2}} \right)$,	(1)

where $\Delta E^{tot}$ is the reaction energy from **Table 1** and ${\Delta\mu}_{O}$ is the chemical potential (referenced to the DFT-computed total energy $E_{O_{2}}^{\mathrm{tot}}$) and $c$ is the stoichiometric coefficient of an oxygen molecule from gaseous oxygen with which the reactants (i.e. LiNiO_2_ and Li_2_ZrO_3_) are in contact. The temperature and pressure dependence of energies of solid phases (e.g. LiNiO_2_, Li_2_ZrO_3_ or Zr-doped LNO) are expected to be much smaller and therefore neglected compared to that of oxygen phase whose chemical potential is given by:

$${\Delta\mu}_{O}\left( T,p_{O_{2}} \right)=\frac{1}{2}\left( \mu_{O_{2}}\left( T,p_{O_{2}} \right)-E_{O_{2}}^{\mathrm{tot}} \right)$$

$=\frac{1}{2}\left( \bar{\mu}_{O_{2}}\left( T,p^{0} \right)+K_{B}T \ln\left( \frac{p_{O_{2}}}{p^{0}} \right) \right)$.	(2)

The standard chemical potential values as function of *T* at 1 atm ($\bar{\mu}_{O_{2}}(T,p^{0})$) were taken from the JANAF thermodynamic tables [10]. It is well known that GGA-PBE functional, which has been used in this study, overbinds the O_2_ molecular energy [11–13]. With respect to the experimental binding energy for an oxygen molecule (E_B,exp_(O_2_):-5.247 eV) [3], the computed O_2_ binding energy (E_B,calc_) has to be corrected by 1.478 eV in the calculation of reaction energies

$E_{B,calc}^{\mathrm{corr}} = E_{B,calc}-E_{B,exp}$.	(3)

The resulting corrected energies $\Delta E_{corr1}^{\mathrm{tot}}$ for reaction (2), (5) and (6) are -33 meV, -25 meV, and -44 meV, respectively. Wang et al [11] has corrected the binding energy of O_2_ molecule to -1.36 eV by comparing experimental and DFT-PBE values of oxide materials such as NiO and Li_2_O with respect to 1/2 O_2_ [12]. We also have corrected the formation energy by applying this proposed correction resulting to a corrected reaction energy $\Delta E_{corr2}^{\mathrm{tot}}$ for reaction (2), (5) and (6) are -33 meV, -24 meV, and -43 meV, respectively. Since the difference between the corrected values using the aforementioned two approaches is small (<3%), hereafter we focus on the corrected values based on Dimitrakis approach. The corrected formation energy based on the method proposed by Wang et al. can be seen in **Figure A2** in SI.

**2. Experimental Section**

**Synthesis of the cathode materials**

Spherical Ni(OH)_2_ and Zr-doped Ni(OH)_2_ precursors were synthesized via a co-precipitation method. For the synthesis of the undoped Ni(OH)_2_ precursor, only NiSO_4_·6H_2_O (Samchun Chemical, Korea) was dissolved in deionized water to prepare a 2 M metal solution. For the synthesis of the Zr-doped Ni(OH)_2_ precursors, NiSO_4_·6H_2_O (Samchun Chemical, Korea) and Zr(SO_4_)_2_·4H_2_O (Daejung Chemical, Korea) were dissolved in deionized water to prepare a 2 M metal solution, with the molar ratio of Ni:Zr=1-*x*:*x* (*x* = 0.01, 0.02, and 0.04) depending on the Zr content. A 17 L batch reactor was filled with deionized water and maintained at 45 °C under a N_2_ atmosphere. A 2 M metal sulphate solution was continuously fed into the reactor, along with 4 M NaOH solution (Samchun Chemical, South Korea) and 10.5 M NH_4_OH solution. The synthesized hydroxide precursors were washed and then dried in a vacuum oven at 110 °C. To synthesize the Zr dry-doped cathode materials, Ni(OH)_2_ precursors were mixed with LiOH·H_2_O (Sigma Aldrich, USA) and ZrO_2_ (Sigma Aldrich, USA) at a molar ratio of Li:(Ni+Zr) = 1.01:1−*x*:*x* (*x* = 0, 0.01, 0.03, and 0.07). For the preparation of Zr wet-doped cathode materials, Zr-doped Ni(OH)_2_ precursors were mixed with LiOH·H_2_O (Sigma Aldrich, USA) at a molar ratio of Li:(Ni+Zr) = 1.01:1.00. The mixtures were then calcined in a tube furnace (Lindberg, Thermo Fisher) at 680 ^o^C for 10 h under an oxygen flow of 2 L min^-1^.

**Material characterization**

The chemical compositions of the cathode materials were determined by inductively coupled plasma–optical emission spectrometry (ICP-OES, iCAP 7000 Series, Thermo Fisher Scientific). The crystal structures of the cathode materials were examined by X-ray diffraction (XRD, Empyrean, PANalytical) using a Cu Kα radiation source and a detector (PIXcel 1D), in reflection geometry, scanning over a 2θ range of 10°–130°. Structural parameters were further determined by full-pattern Rietveld refinement analysis (**Fig. A9**) using the FullProf software [14]. The refinement was carried out through a sequential refinement strategy, in which the fitting parameters were refined stepwise until satisfactory agreement between the observed and calculated diffraction patterns was achieved **(Tab. A7)**. The surface morphologies and cross-sections of the cathode materials were characterized by scanning electron microscopy (SEM, Verios G4UC, FEI) operated at an accelerating voltage of 5 kV. Cross-sections of the samples were prepared using a cross-section polisher (IB-19520CCP, JEOL). Elemental distributions were analyzed through energy-dispersive X-ray spectroscopy combined with SEM (SEM-EDS). For transmission electron microscopy (TEM) analysis, thin specimens were prepared via focused ion beam milling (FIB, Scios, FEI). The local crystal structures of the cathode materials were investigated using transmission electron microscopy (TEM, NeoARM, JEOL) operated at an accelerating voltage of 200 kV and equipped with a cold field emission gun and a 125 nm objective aperture.

**Electrochemical tests**

To examine the electrochemical performance of the cathode materials, cathodes were prepared by mixing the cathode active material, a conductive agent (carbon black, Alfa Aesar), and polyvinylidene fluoride (PVDF, Solet5130, Solvay) in N-methyl-2-pyrrolidone (NMP). The slurry was prepared by mixing the components in a mass ratio of 90:5.5:4.5 with a paste mixer (ARE310, Thinky) and Zr balls. The homogeneously mixed slurry was uniformly coated onto aluminum foil (20 μm thick, UACJ) and dried at 110 ^o^C. The areal loading level was controlled within 4–5 mg cm^-2^. The electrodes were roll-pressed and punched into disks with a diameter of 14 mm. For the half-cell tests, 2032 coin-type half-cells were assembled in an Ar-filled glove box, using polypropylene/polyethylene/polypropylene separators (Celgard 2320, Celgard), lithium metal (Honjo Metal), and an liquid electrolyte consisting of 1.2 M LiPF_6_ dissolved in ethylene carbonate:ethyl methyl carbonate (3:7 in volume ratio) with 2 wt% vinylene carbonate (Dongwha Electrolyte, South Korea). Electrochemical tests were conducted using a battery cycler (TOSCAT-3100U, Toyo System). The cycling performance of the coin-type half-cells was evaluated at a constant C-rate of 0.5 C (90 mA g^-1^), after an activation cycle at 0.1 C (18 mA g^-1^), within the voltage range of 2.7–4.3 V.

**References**

[1]	P.E. Blöchl, ‘Projector augmented-wave method’, *Physical Review B*, vol. 50, Dec. 1994, pp. 17953–17979.

[2]	G. Kresse and J. Furthmüller, ‘Efficient iterative schemes for *ab initio* total-energy calculations using a plane-wave basis set’, *Physical Review B*, vol. 54, Oct. 1996, pp. 11169–11186.

[3]	J.P. Perdew, K. Burke, and M. Ernzerhof, ‘Generalized Gradient Approximation Made Simple’, *Physical Review Letters*, vol. 77, Oct. 1996, pp. 3865–3868.

[4]	J. Heyd, G.E. Scuseria, and M. Ernzerhof, ‘Hybrid functionals based on a screened Coulomb potential’, *The Journal of Chemical Physics*, vol. 118, May. 2003, pp. 8207–8215.

[5]	A.V. Krukau, O.A. Vydrov, A.F. Izmaylov, and G.E. Scuseria, ‘Influence of the exchange screening parameter on the performance of screened hybrid functionals’, *The Journal of Chemical Physics*, vol. 125, Dec. 2006, p. 224106.

[6]	K. Köster, T. Binninger, and P. Kaghazchi, ‘Optimization of Coulomb energies in gigantic configurational spaces of multi-element ionic crystals’, *npj Computational Materials*, vol. 11, Jul. 2025, p. 202.

[7]	Gurobi Optimization, LLC, ‘Gurobi Optimizer Reference Manual’, 2024 https://www.gurobi.com.

[8]	Y. Seto and M. Ohtsuka, ‘*ReciPro* : free and open-source multipurpose crystallographic software integrating a crystal model database and viewer, diffraction and microscopy simulators, and diffraction data analysis tools’, *Journal of Applied Crystallography*, vol. 55, Apr. 2022, pp. 397–410.

[9]	K. Momma and F. Izumi, ‘*VESTA* : a three-dimensional visualization system for electronic and structural analysis’, *Journal of Applied Crystallography*, vol. 41, Jun. 2008, pp. 653–658.

[10]	Thomas C. Allison, ‘NIST-JANAF Thermochemical Tables - SRD 13’, Jan. 2013 https://janaf.nist.gov/. Last accessed on February 26, 2024.

[11]	L. Wang, T. Maxisch, and G. Ceder, ‘Oxidation energies of transition metal oxides within the GGA + U framework’, *Physical Review B*, vol. 73, May. 2006, p. 195107.

[12]	D.A. Dimitrakis, N.I. Tsongidis, and A.G. Konstandopoulos, ‘Reduction enthalpy and charge distribution of substituted ferrites and doped ceria for thermochemical water and carbon dioxide splitting with DFT+U’, *Physical Chemistry Chemical Physics*, vol. 18, 2016, pp. 23587–23595.

[13]	A.S. Rosen, J.M. Notestein, and R.Q. Snurr, ‘Comparing GGA, GGA+ *U* , and meta-GGA functionals for redox-dependent binding at open metal sites in metal–organic frameworks’, *The Journal of Chemical Physics*, vol. 152, Jun. 2020, p. 224101.

[14]	J. Rodríguez-Carvajal, ‘Recent advances in magnetic structure determination by neutron powder diffraction’, *Physica B: Condensed Matter*, vol. 192, Oct. 1993, pp. 55–69.

[15]	H. Kudo, ‘The rates of thermal decomposition of LiOH(s), LiOD(s) and LiOT(s)’, *Journal of Nuclear Materials*, vol. 87, Nov. 1979, pp. 185–188.

[16]	T. Sato, T. Nakamura, and F. Ozawa, ‘Thermal decomposition of nickel hydroxide’, *Journal of Applied Chemistry and Biotechnology*, vol. 25, Aug. 1975, pp. 583–590.

[17]	T. Sato, F. Ozawa, T. Nakamura, H. Watanabe, and S. Ikoma, ‘Thermal decomposition of zirconium hydroxide’, *Thermochimica Acta*, vol. 34, Dec. 1979, pp. 211–220.

[18]	C.A. Strydom and G. Pretorius, ‘The thermal decomposition of zirconium sulphate hydrate’, *Thermochimica Acta*, vol. 223, Aug. 1993, pp. 223–232.

| **Table A3**: ICP-OES results, showing the targeted and the measured incorporated Zr amounts | | |
| --- | --- | --- |
| **Cathode materials** | **Ni**  **(at%)** | **Zr**  **(at%)** |
| Zr 1%-doped LNO | 99.09 | 0.91 |
| Zr 3%-doped LNO | 96.87 | 3.13 |
| Zr 7%-doped LNO | 93.24 | 6.76 |

| **Table A1**: Metal-oxide formation from hydroxide | | | | | | |
| --- | --- | --- | --- | --- | --- | --- |
| **Reactions** | | | | | **Temperature** | **Reference** |
| 2 LiOH | 🡪 | Li_2_O | + | H_2_O | T ~ 150 °C | [15] |
| Ni(OH)_2_ | 🡪 | NiO | + | H_2_O | T = 300 °C | [16] |
| Zr(OH)_4_ | 🡪 | ZrO_2_ | + | 2 H_2_O | T = 140 °C | [17] |
| Zr(OH)_4_ | 🡪 | ZrO_2_ | + | 2 SO_3_ | T = 540 °C | [18] |

| **Table A2**: Calculated reaction energies (in meV per formula unit) for non-doped and doped cases including Li_1-_*_m_*Ni_1+_*_m_*O_2_ with *m*= 1/9, 1/6, 1/5 (as products) using DFT | | |  |
| --- | --- | --- | --- |
| **No** | **Non-doped 🡪 Doped** | ***∆E*^tot^** | |
| (1’) | LiNiO_2_ + 1/99 Li_2_ZrO_3_ → 32/33 Li(Ni_94/96_Li_1/96_Zr_1/96_)_TM_O_2_ + 1/22 Li_8/9_Ni_10/9_O_2_ | −31 | |
| (2’) | LiNiO_2_ + 1/51 Li_2_ZrO_3_ → 16/17 Li(Ni_92/96_Li_2/96_Zr_2/96_)_TM_O_2_ + 3/34 Li_8/9_Ni_10/9_O_2_ | −19 | |
| (3’) | LiNiO_2_ + 1/35 Li_2_ZrO_3_ → 32/35 Li(Ni_90/96_Li_3/96_Zr_3/96_)_TM_O_2_ + 9/70 Li_8/9_Ni_10/9_O_2_ | −9 | |
| (1’’) | LiNiO_2_ + 2/195 Li_2_ZrO_3_ → 64/65 Li(Ni_94/96_Li_1/96_Zr_1/96_)_TM_O_2_ + 2/65 Li_5/6_Ni_7/6_O_2_ | −30 | |
| (2’’) | LiNiO_2_ + 2/99 Li_2_ZrO_3_ → 32/33 Li(Ni_92/96_Li_2/96_Zr_2/96_)_TM_O_2_ + 2/33 Li_5/6_Ni_7/6_O_2_ | −18 | |
| (3’’) | LiNiO_2_ + 2/67 Li_2_ZrO_3_ → 64/67 Li(Ni_90/96_Li_3/96_Zr_3/96_)_TM_O_2_ + 6/67 Li_5/6_Ni_7/6_O_2_ | −6 | |
| (1’’’) | LiNiO_2_ + 1/97 Li_2_ZrO_3_ → 96/97 Li(Ni_94/96_Li_1/96_Zr_1/96_)_TM_O_2_ + 5/194 Li_4/5_Ni_6/5_O_2_ | −30 | |
| (2’’’) | LiNiO_2_ + 1/49 Li_2_ZrO_3_ → 48/49 Li(Ni_92/96_Li_2/96_Zr_2/96_)_TM_O_2_ + 5/98 Li_4/5_Ni_6/5_O_2_ | −18 | |
| (3’’’) | LiNiO_2_ + 1/33 Li_2_ZrO_3_ → 32/33 Li(Ni_90/96_Li_3/96_Zr_3/96_)_TM_O_2_ + 5/66 Li_4/5_Ni_6/5_O_2_ | −6 | |

**Figure A1:** Charge–discharge curves of undoped LNO, as well as Zr dry- and wet-doped LNO cathodes in half-cells.

**Figure A2:** Computed and corrected energy of reactions (2, 5 & 6) as functions of chemical potential of oxygen ($\mu_{O}$), referenced to $E_{O_{2}}^{\mathrm{tot}}.$The temperature dependence for three likely oxygen partial pressure of oxygen is presented on the top.

**Figure A3** Refined a-axis (top) and x-axis (middle) lattice parameter and unit cell volume of LNO and Zr-doped LNO.

**Figure A4:** LNO undoped and and Zr-doped LNO lattice parameters a (top) and c (bottom) from XRD measurements (left) and from simulations (right)

**Figure A5:** Top-, 3d and side view of spherical crosscut-particle models with increasing Zr-concentrations

**Figure A6:** SEM and TEM images of other grains at secondary particle interior and surface.

**A. 1: Calculation of atomic surface and volume ratio and related Zr concentrations**

The total number of atoms of our particle model (N_T,M_), as well as the number of atoms on its surface (N_surf,M_) was determined, as well as its Volume (V_M_) and its surface (A_M_), assumed to be hexagon prism, defined by the edge lengths (a) and the height (h). With that, the Surface/Volume ratio (A/V) was determined with various a and h, while maintaining the a/h ratio from our model (see **Fig**. **A7**). Afterwards the top & bottom surface (A_surf,tb_) and the side surfaces (A_surf,side_) were determined and tother their respective surface atoms (N_surf_), the density of Ni ions $\rho_{Ni}$ can be determined by:

$$\rho_{Ni\left( 0003 \right)}=\frac{N_{Ni,surf}}{A_{surf,tb}}, \rho_{Ni(01\bar{1}2)}=\frac{N_{Ni,surf}}{A_{surf, side}}$$

Afterwards the ratio of atoms on surface versus atoms in volume was calculated by:

$$\frac{N_{\mathrm{surf}}}{N_{\mathrm{volume}}}$$

The now changed Zr-concentrations for the 1μm large particle were assumed by:

$$c_{\mathrm{Zr}}=\frac{N_{Ni,surf,1\mu m}}{N_{Ni ,1\mu m}}\cdot\left( \frac{N_{Zr,M}}{N_{Ni,M}} \right)\frac{N_{\mathrm{surf}}}{N_{\mathrm{volume}}}$$

**Figure A7:** Surface/Volume ratio displayed over different hexagon particle diameters with ● our particle model (d= 69Å) and ● d= 1μm particle.

**Figure A8:** Li_0.75_Ni_1.25_O_2_ as 2x2x1 supercell as 3D (left), along [100] zone (middle) and along [110] zone (right)

**Figure A9.** Rietveld refinement profiles of the XRD patterns for a) LNO, b) Zr 1%-doped LNO, c) Zr 3%-doped LNO, and d) Zr 7%-doped LNO. The observed data (red circles), calculated profiles (black lines), difference curves (blue lines), and Bragg reflection positions (green ticks) are shown.

| **Table A4**: Compositions and their properties used for electrostatic and DFT-PBE calculation | | | | |
| --- | --- | --- | --- | --- |
| **Composition** | **Space group** | **Number of atoms** | **K-Points** | **Cell-size**  **(a,b,c in Å)** |
| Li_2_O | $\mathrm{Fm}\bar{3}m$ | 12 | 4x4x4 | 4.7 x 4.7x 4.7 |
| NiO | $\mathrm{Fm}\bar{3}m$ | 8 | 4x4x4 | 4.2 x 4.2 x 4.2 |
| ZrO_2_ | $P21/c1$ | 12 | 4x4x4 | 4.2 x 4.2 x 4.2 |
| Li_1-x_NiO_2_ | $R\bar{3}m$ | 96 | 5x5x1 | 5.8 x 5.8 x 28.4 |
|  | $R\bar{3}m$ | 108 | 3x3x2 | 8.6 x 8.6 x 14.2 |
|  | $R\bar{3}m$ | 384 | 2x1x2 | 11.5 x 23 x 14.2 |
| Zr-doped LNO  ([Li_1-x_Ni_x_]_AM_[Ni_1-x-z_Li_x_Zr_z_]_TM_O_2_) | $R\bar{3}m$ | 108 | 3x3x2 | 8.6 x 8.6 x 14.2 |
|  | $R\bar{3}m$ | 384 | 2x1x2 | 11.5 x 23 x 14.2 |
| Li_2_ZrO_3_ | $C2/m$ | 24 | 2x1x2 | 5.5 x 9.1 x5.5 |
|  | $C2/m$ | 360 | 1x1x5 | 27.3 x 27.2 x 5.46 |
| Li_1-m_Ni_1+m_O_2_ | $C2/m$ | 24 | 2x1x2 | 5.1 x 8.7 x 5.1 |
|  | $C2/m$ | 360 | 1x1x5 | 25.3 x 26.2 x 5 |

| **Table A5**: Compositions and their properties used for DFT-HSE calculation | | | | |
| --- | --- | --- | --- | --- |
| **Composition** | **Space group** | **Number of atoms** | **K-Points** | **Cell-size**  **(a,b,c in Å)** |
| LiNiO_2_ | $R\bar{3}m$ | 96 | 3x3x1 | 5.8 x 5.8 x 28.4 |
| Zr-doped LNO  (Li_0.96_(Ni_0.92_ Li_0.04_Zr_0.04_)_TM_O_2_) | $R\bar{3}m$ | 108 | 3x3x2 | 8.6 x 8.6 x 14.2 |
| Li_2_ZrO_3_ | $C2/m$ | 24 | 2x1x2 | 5.5 x 9.1 x5.5 |
| Li_0.67_Ni_1.33_O_2_ | $C2/m$ | 24 | 2x1x2 | 5.1 x 8.7 x 5.1 |

| **Table A6**: Compositions and their properties of large-scale particle model used for electrostatic calculation | | |
| --- | --- | --- |
| **Composition** | **Number of atoms** | **Cell-size**  **(a,b,c in Å)** |
| Li_1.03-x_[Ni_0.89-x_Zr_x_]_TM_O_2_  (2336Li 2710Ni 6048O)  with x=0.15, 0.3, 0.37  (400Zr, 800Zr & 1,000Zr) | 11,936 | 121.9 x 121.9 x 98.8 |

**Table A7.** Structural parameters of cathode materials determined by Rietveld refinement of XRD

| LNO | | LiNiO_2_ | | Volume: 101.5872 Å^3^ | | |
| --- | --- | --- | --- | --- | --- | --- |
| *a*-axis: 2.8747 Å | | *c-*axis: 14.1946 Å | |  |  |  |
| Atom | Wyckoff position | x | y | z | B_iso_ | Modified  occupancy (occ*12) |
| Li1 | 3a | 0 | 0 | 0 | 1.000 | 0.99519(1) |
| Ni2 | 3a | 0 | 0 | 0 | 1.000 | 0.00481(1) |
| Ni1 | 3b | 0 | 0 | 0.5 | 0.5000 | 0.99519(2) |
| Li2 | 3b | 0 | 0 | 0.5 | 0.5000 | 0.00481(1) |
| O1 | 6c | 0 | 0 | 0.23988(7) | 0.8000 | 2 |

| LNO + Zr 1% | | Li[Ni_0.991_Zr_0.009_]O_2_ | | Volume: 101.7693 Å^3^ | | |
| --- | --- | --- | --- | --- | --- | --- |
| *a*-axis: 2.8768 Å | | *c-*axis: 14.1993 Å | |  |  |  |
| Atom | Wyckoff position | x | y | z | B_iso_ | Modified  occupancy (occ*12) |
| Li1 | 3a | 0 | 0 | 0 | 1.000 | 0.98878(2) |
| Ni2 | 3a | 0 | 0 | 0 | 1.000 | 0.01122(1) |
| Ni1 | 3b | 0 | 0 | 0.5 | 0.5000 | 0.97978(1) |
| Zr1 | 3b | 0 | 0 | 0.5 | 0.5000 | 0.00900(2) |
| Li2 | 3b | 0 | 0 | 0.5 | 0.5000 | 0.01122(0) |
| O1 | 6c | 0 | 0 | 0.23991(6) | 0.8000 | 2 |

| LNO + Zr 3% | | Li[Ni_0.969_Zr_0.031_]O_2_ | | Volume: 101.8574 Å^3^ | | |
| --- | --- | --- | --- | --- | --- | --- |
| *a*-axis: 2.8777 Å | | *c-*axis: 14.2027 Å | |  |  |  |
| Atom | Wyckoff position | x | y | z | B_iso_ | Modified  occupancy (occ*12) |
| Li1 | 3a | 0 | 0 | 0 | 1.000 | 0.97059(3) |
| Ni2 | 3a | 0 | 0 | 0 | 1.000 | 0.02941(1) |
| Ni1 | 3b | 0 | 0 | 0.5 | 0.5000 | 0.93959(1) |
| Zr1 | 3b | 0 | 0 | 0.5 | 0.5000 | 0.03100(2) |
| Li2 | 3b | 0 | 0 | 0.5 | 0.5000 | 0.02941(2) |
| O1 | 6c | 0 | 0 | 0.23991(7) | 0.8000 | 2 |

| LNO + Zr 7% | | Li[Ni_0.932_Zr_0.068_]O_2_ | | Volume: 101.9887 Å^3^ | | |
| --- | --- | --- | --- | --- | --- | --- |
| *a*-axis: 2.8792 Å | | *c-*axis: 14.2062 Å | |  |  |  |
| Atom | Wyckoff position | x | y | z | B_iso_ | Modified  occupancy (occ*12) |
| Li1 | 3a | 0 | 0 | 0 | 1.000 | 0.95098(2) |
| Ni2 | 3a | 0 | 0 | 0 | 1.000 | 0.04902(1) |
| Ni1 | 3b | 0 | 0 | 0.5 | 0.5000 | 0.88298(1) |
| Zr1 | 3b | 0 | 0 | 0.5 | 0.5000 | 0.06800(2) |
| Li2 | 3b | 0 | 0 | 0.5 | 0.5000 | 0.04902(3) |
| O1 | 6c | 0 | 0 | 0.23991(5) | 0.8000 | 2 |

1. ^+^ These authors contributed equally to this study.
2. Δ Present address of M. Ihrig: AUDI AG, NSU-Str. 1, 74172 Neckarsulm, Germany
3. ^*^ Corresponding authors: [yksun@hanyang.ac.kr](mailto:yksun@hanyang.ac.kr), [p.kaghazchi@fz-juelich.de](mailto:p.kaghazchi@fz-juelich.de)
